# Integrated Proteomic and Metabolomic Analyses Characterise Molecular Alterations Associated with JSRV-Induced OPA

**DOI:** 10.3390/biology15130982

**Published:** 2026-06-23

**Authors:** Pei Zhang, Xujie Duan, Yu Wang, Anyu Bao, Xinqi Ma, Sixu Chen, Yufei Zhang, Shuying Liu

**Affiliations:** 1 College of Veterinary Medicine, Inner Mongolia Agricultural University, Hohhot 010018, China; 15149366412@163.com (P.Z.); 15344155849@163.com (Y.W.); 15848106411@163.com (A.B.); 15561394939@163.com (X.M.); chans1001@163.com (S.C.); enjoy_zyf@163.com (Y.Z.); 2 Inner Mongolia Key Laboratory of Veterinary Fundamentals and Disease Control of Herbivorous livestock, Inner Mongolia Agricultural University, Hohhot 010018, China; 3 Inner Mongolia Key Laboratory of Basic Veterinary Medicine, Inner Mongolia Agricultural University, Hohhot 010018, China

**Keywords:** ovine pulmonary adenocarcinoma, Jaagsiekte sheep retrovirus, metabolomics, proteomics

## Abstract

Ovine pulmonary adenocarcinoma (OPA), caused by the exogenous Jaagsiekte sheep retrovirus (JSRV), shares several pathological and molecular features with human lung adenocarcinoma, providing an important model for comparative oncology. JSRV pathogenesis is mostly studied at the transcriptome level, with systematic proteomic and metabolomic studies remaining insufficient. Therefore, this study aimed to systematically characterise the molecular alterations associated with JSRV-induced OPA by integrating direct data-independent acquisition proteomics and untargeted metabolomics. We established an OPA model by infecting lambs with JSRV and performed multi-omics analyses on the lesion and control lung tissues (*n* = 3 per group for both proteomic and metabolomic analyses). In total, 1631 differentially expressed proteins and 748 differential metabolites were identified, and the two omics datasets exhibited highly coordinated variations. Integrated analyses suggested that adhesion remodelling (with downregulated LIMCH1), endoplasmic reticulum stress (with upregulated HYOU1), and metabolic and immune-related alterations (accompanied by elevated GFPT1 and PTGS2) represent major biological processes associated with JSRV infection. Western blot analysis confirmed the expression changes in these proteins. Overall, this study provides a multidimensional molecular landscape of OPA and expands current understanding of the molecular alterations associated with JSRV infection. These findings provide candidate pathways and molecular targets for future mechanistic studies and comparative investigations of lung adenocarcinoma.

## Simple Summary

Ovine pulmonary adenocarcinoma (OPA), an infectious lung tumour caused by the ovine pulmonary adenocarcinoma virus (JSRV), causes severe economic losses and welfare issues in the global sheep industry and provides an important model in comparative oncology due to its high similarity with human lung adenocarcinoma in terms of pathological features, cell origin, and carcinogenic pathways. Although the pathogenic mechanisms of JSRV at the transcriptome level have been studied, systematic analyses at the protein and metabolic levels are needed to understand the mechanisms underlying OPA occurrence and development. Here, we integrated proteomic and metabolomic analyses to analyse the molecular expression patterns between JSRV-induced OPA lesions and healthy lung tissues and the coordinated regulatory features. We found that JSRV infection can drive global protein and metabolic profile remodelling in host lung tissues and identified three core pathogenic molecular programmes of adhesion remodelling, endoplasmic reticulum stress, and metabolic reprogramming, and immune activation. We also screened and validated key regulatory molecules such as GFPT1, PTGS2, and ARG1. Overall, this study is the first to map the pathogenic protein-metabolic synergy network of OPA from a multidimensional molecular perspective. It may help clarify the tumourigenic mechanism of retroviruses and facilitate screening of potential therapeutic targets for human lung adenocarcinoma.

## 1. Introduction

Ovine pulmonary adenocarcinoma (OPA) is an infectious pulmonary neoplastic disease caused by exogenous Jaagsiekte sheep retrovirus (JSRV). It causes significant animal welfare issues and economic losses in the sheep industry worldwide [1]. Clinically, the affected sheep often present progressive dyspnoea, weight loss, and decreased exercise tolerance. In the late stage of the disease, a large volume of fluid accumulates in the lungs and flows out of the nostrils when the head is lowered [2]. The subclinical period of OPA is typically long, and once clinical symptoms appear, the lung tumours have often spread extensively, leading to a fatality rate of nearly 100%. Additionally, secondary bacterial or parasitic infections often occur in the later stages of the disease and exacerbate its progression.

JSRV belongs to the β-retrovirus family, and its envelope glycoprotein Env can directly induce cell transformation and lung tumour formation both in vitro and in vivo [3,4,5]. This virus has a clear tropism for differentiated distal lung epithelial cells, and OPA tumour cells mainly express markers related to type II alveolar epithelial cells (AT2) [6]. Previous studies have confirmed that JSRV Env activates multiple signalling pathways related to cell proliferation and survival, including the PI3K/Akt and MAPK/ERK pathways. Further, several host factors are involved in the Env-mediated cell transformation process [7,8]. However, despite the clear identification of JSRV as the causative agent of OPA, the molecular events from viral infection to tumour development, tissue remodelling, and formation of the characteristic clinical phenotype have not been fully characterised.

Notably, infected sheep can only generate a limited adaptive immune response to JSRV antigens, which hinders the development of effective serological diagnostic methods and vaccines [9,10]. This weak immune response is at least partially related to the immune tolerance induced by the expression of endogenous JSRV (enJSRV)-related proteins during foetal thymus development in sheep [11,12]. The local immune regulatory mechanism in the lungs of OPA-affected sheep also promotes virus persistence and tumour progression [13]. After JSRV infection in sheep, immune cell phenotypes are altered, accompanied by robust macrophage infiltration and partial immune dysfunction, suggesting that OPA develops in a unique tumour-associated immune microenvironment [13].

Owing to the special nature of OPA in veterinary clinical practice and tumour occurrence, OPA has received increasing attention in recent years because of its similarities to human lung adenocarcinoma in terms of histopathology, cell origin, and oncogenic signalling pathway activation, being regarded as a naturally occurring comparative oncology model [14,15]. In the early disease stages, OPA can exhibit characteristics of minimally invasive adenocarcinoma with predominant adherent growth, whereas in the advanced stages, these lesions are more similar to those of papillary or acinar adenocarcinoma, and some cases may also have mucinous features [16,17]. Compared with traditional rodent models, OPA has higher translational potential in terms of imaging assessment, longitudinal dynamic monitoring, interventional operations, and evaluation of devices or local treatment strategies [17,18]. These characteristics also make OPA an important large animal model for studying lung tumour biology and bridging the gap between experimental oncology and clinical application [16,17].

Recent research on the host response to JSRV infection has gradually expanded to the transcriptomic level. RNA sequencing of lung tissues from experimentally infected lambs revealed that JSRV infection leads to extensive changes in host gene expression, involving multiple processes, such as tumourigenesis, epithelial differentiation, cytokine and chemokine signalling, complement activation, and macrophage-related pathways [13]. Further, the AGR2-YAP1-AREG axis is activated in OPA tumour cells, further confirming the similarity between OPA and human lung adenocarcinoma, especially early-stage lung adenocarcinoma [13]. However, transcriptome information alone cannot fully reflect the functional state of the lesion tissue. Proteins execute biological functions encoded by genes, and metabolites act downstream of protein regulatory networks. Quantitative analysis of proteins and metabolites can dissect the complex interrelationships among genes and the microenvironment, thereby revealing a large number of “translatable” diagnostic and therapeutic targets. Furthermore, transcript abundance does not necessarily correlate with functional protein levels because of post-transcriptional regulation, protein turnover, and dynamic metabolic adaptation. Proteomics directly reflects changes in functional protein expression, whereas metabolomics captures the terminal biochemical consequences of cellular processes. Therefore, integrating proteomic and metabolomic data provides complementary biological information and enables a more comprehensive characterisation of disease-associated molecular alterations than either approach alone. Therefore, to fully understand the pathogenesis of OPA, research perspectives must expand beyond transcriptome analysis.

To address the current lack of systematic evidence at the protein and metabolic levels in OPA, we established an OPA model using lambs experimentally infected with JSRV, selected typical lung lesions and the corresponding healthy control tissues from the same anatomical sites, and conducted direct data-independent acquisition (DIA) proteomics and non-targeted metabolomics detection. Common pathways and potential hub molecules were identified using enrichment analyses, interaction networks, and cross-omics integration models. Specifically, this study was designed to address the following biological question: can integrated proteomic and metabolomic analyses systematically characterise the molecular alterations associated with JSRV-induced OPA and identify candidate pathways potentially involved in disease progression? The aim of this study was to systematically characterise cross-omics molecular alterations in OPA lesion tissues and to identify prioritised candidate pathways, key proteins, and metabolites for subsequent mechanistic studies and targeted validation. To enhance the reliability of the results, we further verified key differentially expressed proteins using Western blotting.

## 2. Materials and Methods

### 2.1. Establishment of the Experimental OPA Model and Collection of Lung Tissues

The JSRV plasmid was transfected into 293T cells using Lipofectamine 3000 (Thermo Fisher Scientific, Waltham, MA, USA) to rescue JSRV [19]. Cell supernatants were collected at 24, 48, and 72 h post-transfection, and then centrifuged and filtered through a 0.45 μm membrane (MilliporeSigma, Darmstadt, Germany) to obtain the virus suspension. The viral titre was quantified, and the inoculation dose was determined using an HIV reverse transcriptase (RT) assay (Roche colorimetric Reverse Transcriptase Assay, Merck, Gillingham, UK). Six 6-day-old Suffolk male lambs were randomly divided into a JSRV infection group and a negative control group (*n* = 3 per group). The infection group was intratracheally inoculated with a JSRV suspension containing 2000 ng of HIV RT activity, whereas the control group was inoculated with the same volume of PBS [20]. The lambs were continuously observed clinically until they showed the typical respiratory symptoms and then euthanised. The OPA model was confirmed to be successfully established via pathological examination. Multimodal analysis was performed using lung tissues from the experimental animals. The typical lesion area of the lungs was taken from the infection group, whereas healthy lung tissues at the corresponding anatomical sites were collected from the control group. Histopathological evaluation, including HE staining assessment and JSRV Env immunohistochemical interpretation, was performed by investigators blinded to treatment group allocation. Proteomic analysis was performed using three independent biological replicates per group, with one representative tissue sample collected from each lamb. For metabolomic analysis, each group also consisted of three biological replicates based on individual lambs. To account for potential spatial heterogeneity within tumour lesions, two anatomically distinct regions were sampled from each lamb, generating six technical subsamples per group. These subsamples were used to improve detection robustness and reduce sampling bias but were not treated as independent biological replicates in statistical analyses. All samples were rinsed with pre-cooled sterile saline, rapidly frozen in liquid nitrogen, and stored at −80 °C until analysis.

### 2.2. Proteomics Analysis

The proteomic analysis was conducted by Nanjing Paisonno Gene Technology Co., Ltd. The frozen lung tissues were processed by lysis, protein extraction, BCA quantification, reduction and alkylation, enzymatic digestion with trypsin, desalination, and drying. A Vanquish Neo UHPLC system combined with an Orbitrap Astral mass spectrometer (Thermo Fisher Scientific, Bremen, Germany) was used to perform liquid chromatography-tandem mass spectrometry analysis in the Direct DIA mode.

The original proteomic sequencing data were imported into the DIA-NN (v1.9.2) software. The prediction library generated by deep learning was used for a direct spectral library-free search and quantification. The *Ovis aries* protein database was used as a reference. Finally, the protein expression matrix was generated. Each proteomic group contained three biological replicates without technical replicates. Considering the relatively small sample size (*n* = 3), the assumptions required for parametric statistical testing could not be reliably satisfied. Therefore, differential protein expression analysis was performed using the non-parametric Wilcoxon rank-sum test. To control the false discovery rate arising from multiple comparisons, *p*-values were adjusted using the Benjamini–Hochberg method. Proteins with |log2(Fold Change)| > 1 and FDR < 0.05 were considered differentially expressed proteins (DEPs). Functional enrichment analyses of DEPs were subsequently conducted using Gene Ontology (GO) and Kyoto Encyclopedia of Genes and Genomes (KEGG) databases.

### 2.3. Metabolomics Analysis

Metabolites from frozen lung tissues were extracted using a pre-cooled methanol–acetonitrile–water mixture. After grinding, ultrasonic treatment, centrifugation, drying, and filtration, LC-MS detection was performed using an ACQUITY UPLC system in conjunction with a Thermo Orbitrap Exploris 120 mass spectrometer (Thermo Fisher Scientific, Bremen, Germany) in the positive and negative ion modes. Quality control samples were prepared using equal volumes of mixed samples, and data stability was monitored throughout the process. The raw data were preprocessed using Compound Discoverer 3.3 software, including peak detection, peak alignment, deconvolution, and compound annotation. Metabolite identification was performed by matching accurate mass, isotope distribution, and MS/MS fragmentation spectra against public databases, including the Human Metabolome Database (HMDB). Features that could not be confidently annotated were excluded from downstream biological interpretation. After filtering out characteristic peaks with an RSD of less than 30%, the Wilcoxon rank-sum test combined with OPLS-DA analysis was used to screen the differentially expressed metabolites. The robustness and predictive performance of the OPLS-DA model were evaluated using permutation testing to assess potential model overfitting. The screening criteria were |log2(FC)| > 1, VIP > 1, and FDR < 0.05. Finally, KEGG pathway enrichment analysis was performed for the differentially expressed metabolites.

### 2.4. Joint Analysis of Proteomic and Metabolomic Data

After screening for differentially expressed proteins and metabolites, the datasets were matched according to the corresponding biological samples and subjected to unit variance (UV) scaling prior to integrative analysis. Principal component analysis (PCA) was first performed to evaluate the overall structure of the integrated dataset. O2PLS analysis was subsequently performed using the OmicsPLS package (v2.0.0) in R (v4.3.2) to explore the global association between the proteomic and metabolomic profiles. Model quality was evaluated using the explained variance parameters R2X, R2Y, R2X(corr), and R2Y(corr) to assess the reliability and interpretability of the model. Given the exploratory nature and limited sample size of this study, the O2PLS analysis was primarily used to characterise global covariance patterns between the two omics datasets rather than for predictive modelling. KEGG enrichment analysis was separately performed on the differentially expressed molecules, and the common significantly enriched pathways were defined as “common pathways”. For the key molecules within the common pathways, the Spearman rank correlation coefficient was calculated, with |r| > 0.8 and *p* < 0.05 as the threshold to screen significant correlations, and a protein–metabolite-related network was constructed to analyse the potential regulatory connections within the pathways.

### 2.5. Western Blot Verification

Three representative differentially expressed proteins (GFPT1, PTGS2, and ARG1) identified by proteomic analysis were selected for Western blot validation to confirm the consistency of protein expression changes observed in the proteomic dataset. After extracting the total protein from the lung tissues, it was separated using SDS-PAGE and transferred onto a PVDF membrane. The membrane was then blocked and successively incubated with primary and secondary antibodies. Colour development and image acquisition were performed using chemiluminescence. The grey values of the bands were analysed using ImageJ software (1.48v, National Institutes of Health, Bethesda, MD, USA), and the expression level of the target protein was represented as its normalised intensity relative to the internal reference protein.

### 2.6. Statistical Analysis

Unless otherwise specified, statistical analysis and graph plotting were conducted using GraphPad Prism 9.3.0 and R language. Given the small sample size used for proteomics and the difficulty in ensuring a normal distribution, group differences between the infection and control groups were primarily tested using the non-parametric Wilcoxon rank-sum test, and multiple test corrections (FDR) were performed using the Benjamini–Hochberg method. For Western blot verification, if the data met the conditions of normality and homogeneity of variance, unpaired *t*-tests were used; otherwise, the Mann–Whitney U test was employed. Statistical significance was set as *p* < 0.05.

## 3. Results

### 3.1. Successful Establishment of the OPA Model

#### 3.1.1. Clinical Symptoms

After the artificial challenge, the infected group of lambs exhibited significant individual differences in respiratory symptoms: Sheep No. 5 developed persistent coughing and abdominal breathing, which are typical clinical symptoms of OPA, 122 days after the challenge; Sheep No. 4 and 6 showed the above typical respiratory symptoms 155 days after the challenge, whereas the negative control group of lambs showed no abnormal clinical symptoms throughout the process.

#### 3.1.2. Gross Pathological Observation

The gross autopsy results showed that the lesions were confined to the respiratory system. In the infected group, no abnormalities were observed in the hearts, livers, spleens, kidneys, or lymph nodes of the sheep. The representative pathological results are shown in Figure 1. In the negative control group (sheep No. 1) the surface of the lung tissue was smooth, the texture was soft, and the colour was normal, with no visible lesions under the naked eye (Figure 1A,C); no foreign substances were found in the trachea (Figure 1B); the lung section was flat, without nodules, consolidation foci, or abnormal fluid exudation (Figure 1D). In the JSRV-infected group (sheep No. 5) the lungs were significantly enlarged, with a hard texture, and scattered grey-white nodules and diffuse consolidation were visible on the surface (Figure 1E,G); the trachea was filled with white foamy mucus (Figure 1F); greyish-white sand-grain-like protruding nodules were visible on the lung section (Figure 1H). These gross lesions were consistent with the typical pathological features of ovine pulmonary adenocarcinoma (OPA), confirming that successful JSRV infection induced the occurrence of OPA.

#### 3.1.3. Haematoxylin and Eosin and Immunohistochemical Staining

The haematoxylin and eosin staining results are shown in Figure 2. In the negative control group (Sheep No. 1), no obvious pathological changes were observed in the lung tissue (Figure 2A,B), which presented a complete alveolar structure, well-expanded alveolar cavities, no thickening of the alveolar septa, and a regular morphology and arrangement of alveolar epithelial cells. In contrast, the lung tissue of the experimental group (Sheep No. 5) showed typical OPA lesion characteristics (Figure 2C,D), including significant proliferation of alveolar type II epithelial cells, presenting as cuboidal or columnar cells, protruding into the alveoli, forming typical adenocarcinoma foci (red arrows), along with a large number of macrophages infiltrating the alveolar cavities. Immunohistochemical analysis of lung tissue from the JSRV infection group showed positive reactions for JSRV Env in the diffuse tumorous epithelial cells. Figure 2C,D showed that JSRV Env showed strong positive brown staining in the cytoplasm of alveolar type II epithelial cells (black arrows), whereas no positive signal was observed in the negative control group (Figure 2(Aa,Bb)); these pathological features were consistent with the diagnostic criteria for OPA [14,21].

### 3.2. Differential Proteomic Analysis

#### 3.2.1. Proteomics Identification Results and Data Quality Assessment

Based on the Direct DIA quantitative technology, the lung tissues of sheep in the JSRV infection and negative control groups were subjected to protein mass spectrometry analysis. After searching the sheep UniProt reference database, 98,746 peptide segments and 8611 proteins were identified. Among these, 8429 proteins were suitable for quantitative analysis and met the requirements for subsequent analysis. Through quality control, we confirmed appropriate enzyme digestion, chromatographic separation, and quantitative stability, with high intra-group sample repeatability and reliable data (detailed quality control data can be found in Supplementary Figure S1).

#### 3.2.2. Overall Protein Expression Patterns of the JSRV and Control Groups Were Significantly Separated

To clarify the overall differences in protein expression profiles between the two groups, we conducted correlation analysis, PCA, hierarchical clustering, and Venn diagram construction. The results showed that the intra-group samples of the two groups were consistent (Figure 3A), the overall protein expression patterns were significantly separated (Figure 3B), and could be clearly clustered according to the groups (Figure 3C). There were a total of 8022 proteins in the two groups, and the specific proteins unique to the JSRV and NC groups were 253 and 154, respectively (Figure 3D), confirming that JSRV infection can drive a systematic change in the protein profile of the host lung tissue.

#### 3.2.3. Identification of Differentially Expressed Proteins

Based on the criteria |log2 (FC)| > 1 and FDR < 0.05, 1631 differentially expressed proteins (DEPs) were identified (DEPs upregulated: 686; downregulated: 945; Figure 4A). The hierarchical clustering heatmap of the differential proteins showed that the samples of the JSRV and NC groups could be clearly distinguished, and the expression patterns within the groups were consistent, confirming that JSRV infection accompanies significant remodelling of the protein profile in the lung tissue (Figure 4B). Further clustering analysis of the top 50 DEPs revealed that the two groups of samples could be clearly distinguished and that most of the top differential proteins in the JSRV group showed a downward trend (Figure 4C). Combined with the analysis of protein functional annotations, these downregulated proteins are mainly involved in biological processes such as cytoskeleton assembly and adhesion regulation (LIMCH1, TNS1, ITGB1, etc.), metabolism and oxidative stress homeostasis maintenance (FABP1, GGT1, CAVIN3, etc.), and key kinase anchoring and proliferation-related signal cascade transmission (AKAP2, ARMC6, etc.), suggesting that JSRV infection can drive cytoskeletal remodelling in the lung tissue, metabolic homeostasis changes, and proliferation-related signal network perturbations.

#### 3.2.4. GO Enrichment Analysis of Differential Proteins

Based on the functional clues of the TOP differential proteins, we conducted GO functional enrichment analysis on all the differential proteins and selected the top 30 entries for analysis to systematically analyse the biological functions of the differentially expressed proteins induced by JSRV infection (Figure 5A). The differentially expressed proteins were significantly enriched in three major aspects: biological process (BP), cellular component (CC), and molecular function (MF). The core aspects indicated adhesion remodelling and endoplasmic reticulum stress. In the BP aspect, significant enrichment was observed for core entries such as cell adhesion, integrin-mediated signalling pathways, and actin cytoskeleton organisation; in the CC aspect, adhesion and scaffold-related structures such as focal adhesions and stress fibres were mainly enriched, while the endoplasmic reticulum–Golgi complex-related membrane structures also showed significant enrichment, suggesting abnormal intracellular endoplasmic reticulum homeostasis and activation of stress responses; in the MF aspect, the core enriched entries were muscle protein binding. Overall, JSRV infection may regulate muscle protein binding function, systematically restructure the cell adhesion-related structures, drive core protein-level changes in adhesion remodelling, and activate intracellular endoplasmic reticulum stress. Enrichment of secondary pathways such as blood coagulation and protein translation also suggests that related processes are jointly involved in OPA disease progression.

#### 3.2.5. KEGG Enrichment Analysis of Differential Proteins

Based on the GO enrichment results, we conducted a KEGG pathway enrichment analysis of the differentially expressed proteins to further analyse the molecular regulatory mechanism of JSRV infection (Figure 5B). The results showed that 76 of the 338 annotated pathways were significantly enriched (*p* < 0.05), and the enrichment features were highly consistent with those of the GO analysis. The core was mainly concentrated in three pathways: adhesion remodelling, immune inflammatory activation, and endoplasmic reticulum stress. Among these, the adhesion remodelling-related pathway was the most significantly enriched. ECM–receptor interaction, focal adhesion, and cell adhesion molecules were highly enriched pathways, directly corresponding to entries such as cell adhesion and actin cytoskeleton organisation in GO analysis, confirming that JSRV infection can drive the systematic reconfiguration of cell adhesion and the cytoskeleton. Simultaneously, immune inflammation-related pathways such as complement and coagulation cascade reactions, leukocyte transendothelial migration, and endoplasmic reticulum protein processing were significantly enriched, suggesting that infection reconfigures the pulmonary immune microenvironment and activates intracellular endoplasmic reticulum stress. Enrichment of the alanine/aspartate/glutamate metabolism pathway also reflected the abnormal amino acid metabolism accompanying the infection. Pathway interaction network analysis showed that these core pathways formed an associated functional network through shared differential proteins (Figure 5C), suggesting that adhesion remodelling, immune activation, endoplasmic reticulum stress, and metabolic reprogramming participate synergistically in the pathogenic process of JSRV infection.

#### 3.2.6. Protein–Protein Interaction Analysis

Based on the GO and KEGG enrichment results, we constructed a protein–protein interaction (PPI) network using the DEPs to identify the key regulatory hubs involved in JSRV infection (Figure 5D). A total of 36 strong interaction core proteins were screened, and their functions were concentrated in the two modules of endoplasmic reticulum stress regulation and metabolic inflammation-coordinated regulation. Among these, HYOU1, SSR1, and SSR3 are the core molecules of endoplasmic reticulum stress, whereas ASS1, GFPT1, PTGS2, and ARG1 are the core nodes of metabolic inflammation regulation. Thus, JSRV infection can synergistically drive the abnormal remodelling of metabolic and inflammatory pathways through these core molecules, promoting the malignant transformation of lung epithelial cells and tumour occurrence.

### 3.3. Differential Metabolomic Analysis

#### 3.3.1. Quality Control Assessment and Differential Metabolite Screening and Chemical Classification

To investigate the JSRV infection-associated metabolic changes, we conducted a non-targeted metabolomic analysis of lung tissues from the two groups of sheep. Quality control verification indicated stable instrument operation and good intra-group sample repeatability. The overall metabolic profiles between the two groups were significantly different and the data were reliable (Figure 6A,B, Supplementary Figure S2). Univariate analysis revealed extensive metabolic differences between the two groups. Strict screening identified 748 key differential metabolites, including 336 upregulated and 412 downregulated metabolites. The two groups of samples were clearly distinguished by differential metabolites through clustering (Figure 6C). The metabolite annotation results revealed a total of 1215 metabolites, with lipids and lipid molecules accounting for the highest proportion, suggesting that lipid remodelling is the core feature of the JSRV infection (Figure 6D).

#### 3.3.2. Identification and Clustering of Differential Metabolites

To assess the metabolic differences between the two groups, we conducted OPLS-DA analysis, which revealed significant differences in the metabolic profiles between the JSRV and NC groups (Figure 7A); the permutation test confirmed the model stability with no overfitting (Q^2^ = 0.865, post-permutation Q^2^ = 0.04, Figure 7B). Based on the criteria of VIP > 1 and Q value < 0.05, 748 key differential metabolites were obtained (upregulated 336, downregulated 412), and hierarchical clustering analysis showed that the samples from the two groups were clearly distinguished by the differential metabolites, confirming their high correlation with JSRV infection (Figure 7C).

#### 3.3.3. KEGG Enrichment Analysis of Differential Metabolites

To further analyse the functional regulation mediated by the differential metabolites, we conducted a KEGG pathway enrichment analysis (Figure 7D). The enrichment characteristics of the differential metabolites were highly consistent with the proteomic results, mainly focusing on three major pathways: metabolic transport, inflammation-related metabolism, and tumour-related metabolism. Among these, pathways such as ABC transporters, protein digestion and absorption, and amino acid biosynthesis showed highly significant enrichment, suggesting that JSRV infection is associated with systemic alterations in amino acid metabolism and metabolite transport in lung tissues. Significant enrichment of the arachidonic acid metabolism pathway indicates that infection is accompanied by inflammation-related metabolic disorders, and enrichment of the carbon metabolism pathway in the cancer centre suggested the presence of metabolic reprogramming-related alterations in lung tissues following JSRV infection. Pathways such as serotonergic synapses and neural activity ligand–receptor interactions were also significantly enriched, suggesting a potential association between infection and metabolic processes related to neuroimmune regulation in lung tissue, providing a core clue for analysing JSRV infection-mediated metabolic disorders.

### 3.4. Joint Analysis of the Protein and Metabolism Groups

To explore the coordinated molecular regulatory characteristics at different omics levels, we conducted a multi-omics integration analysis by combining the protein and metabolic group data. PCA results of the integrated dataset showed a clear distinction between the JSRV infection and negative control (NC) groups, and samples in the same group were clustered closely. This confirmed that the integrated dataset effectively reflected the differences in molecular expression between groups (Figure 8A). Furthermore, the O2PLS model was used to analyse the cross-omics covariance association between the two omics methods. The overall degree of fit of the model was good, with explanatory rates of the independent variable R2X = 0.968 and the explanatory rate of the dependent variable R2Y = 0.878. After correction, the correlation parameters R2X (corr) = 0.953 and R2Y (corr) = 0.823 were obtained, suggesting substantial covariance between the proteomic and metabolomic datasets and indicating that JSRV infection is associated with coordinated alterations in protein expression and metabolite abundance (Figure 8B).

#### 3.4.1. Common Pathway Enrichment Analysis

To identify the pathways that underwent synchronous changes at both the protein and metabolic levels, we compared the KEGG enrichment results of the two omics methods. Venn analysis revealed that 140 pathways were commonly enriched, accounting for 40.7% of all enriched pathways. These included 197 protein-specific enriched pathways and seven metabolic-specific enriched pathways, suggesting that JSRV infection triggered a coordinated perturbation of shared biological programmes (Figure 8C). Common enrichment pathway analysis showed that the core common pathways included arachidonic acid metabolism, alanine/aspartate/glutamate metabolism, glutathione metabolism, glycine/serine/threonine metabolism, and folate-mediated one-carbon unit metabolism, collectively indicating integrated changes in inflammatory lipid signalling, amino acid metabolism, redox regulation, and stress-related cellular processes (Figure 8D).

#### 3.4.2. Analysing Potential Interaction Links Between Proteins and Metabolites Through Network Analysis

To further elucidate the molecular regulatory relationships within the co-enriched pathways, we constructed a correlation network based on the key proteins and metabolites of the core pathways (Figure 8E). Using a threshold of ∣r∣ > 0.8 and *p* < 0.05, we identified several significant protein–metabolite associations wherein negative correlations were dominant with some positive correlations. Among the key proteins, GFPT1, PTGS2, NIT2, and CAMK2 were negatively correlated with multiple metabolites; GFPT1 was negatively correlated with 1-stearoyl lysophosphatidylcholine and citrate; ASL and NIT2 were negatively correlated with succinate, and CAMK2 was negatively correlated with 1-stearoyl lysophosphatidylcholine; RAC1 was positively correlated with 1-stearoyl lysophosphatidylcholine and prostaglandin E2 (PGE2); and PTGS2 had a complex association pattern, negatively correlated with PGE2 and positively correlated with succinate and 1-stearoyl lysophosphatidylcholine. Notably, although PTGS2 was upregulated, it was negatively correlated with its classical metabolite PGE2, indicating an inverse association between PTGS2 abundance and PGE2 levels, the biological basis of which remains unclear.

In summary, succinate, PGE2, and 1-stearoyl lysophosphatidylcholine are key metabolic nodes connecting the protein group remodelling mediated by JSRV infection and metabolic changes. These observations suggest that alterations in key proteins may be associated with changes in metabolic homeostasis during JSRV infection, ultimately causing abnormal metabolism in the lung tissue.

### 3.5. Confirmation of Key Protein Regulation by Western Blot Analysis

To validate the proteomics results, we selected three key proteins, GFPT1, PTGS2, and ARG1, from the PPI network for Western blot detection. Western blot bands (Figure 8F) and relative expression analysis (Figure 8G) showed that, compared with the NC group, the JSRV group showed significantly elevated expression levels of these three proteins (GFPT1: *p* < 0.01; PTGS2: *p* < 0.05; ARG1: *p* < 0.001), consistent with the quantitative results of proteomics. This result supports the reliability of the proteomics data and confirms the upregulation of these candidate proteins in JSRV-infected lung tissues.

## 4. Discussion

As an oncogenic retrovirus, JSRV induces OPA, which is highly similar to human lung adenocarcinoma in terms of pathological histology, imaging features, and oncogenic pathways, providing significant value for translational medical research [14,15]. The pathogenic mechanism of JSRV is mainly studied at the transcriptome level; however, owing to the influence of post-transcriptional regulation, translation efficiency, and protein degradation, the changes in transcript, protein, and metabolite levels are not fully consistent. Simple transcriptome sequencing cannot fully reveal the host molecular regulation and functional changes mediated by viral infection. This study is the first to combine Direct DIA proteomics and non-targeted metabolomics to systematically map the protein and metabolic molecular profiles of JSRV-infected lung tissues, overcoming the limitations of single-omics studies and revealing that JSRV infection is associated with widespread molecular alterations in lung tissue. Cell adhesion remodelling, endoplasmic reticulum stress, and metabolic reprogramming appear to represent three major molecular programmes associated with JSRV infection and may interact with each other during disease progression.

Cell adhesion remodelling is the most prominent proteomic feature of JSRV infection. Functional annotation of the top 50 differentially expressed proteins showed that the most significantly downregulated proteins in the JSRV-infected group were concentrated in cytoskeleton assembly and cell adhesion regulation pathways, including LIMCH1, TNS1, and ITGB1. Downregulation of these proteins may be associated with early changes in epithelial cell polarity and migratory potential [22], highly consistent with the existing research conclusions; for instance, low LIMCH1 expression in lung cancer increases the risk of poor prognosis through interaction with the LRIG protein [23]; ITGB1 promotes tumour proliferation by activating the PI3K/AKT pathway [24]; in non-small cell lung cancer and renal cell carcinoma, low TNS1 expression disrupts epithelial cell adhesion homeostasis and may be associated with epithelial–mesenchymal transition (EMT) and early malignant transformation [25,26,27], consistent with the significant downregulation of TNS1 in the JSRV-infected group in this study.

GO analysis showed that the differentially expressed proteins are significantly enriched at the biological process level in cell adhesion, integrin-mediated signalling pathways, and actin cytoskeleton organisation; at the cellular component level in focal adhesions and stress fibres; and at the molecular function level with actin binding as the core enriched item (Figure 5A); KEGG analysis showed significant enrichment of the ECM–receptor interaction, focal adhesion, and cell adhesion molecule pathways, consistent with the GO results (Figure 5B). Multiple lines of evidence from protein and pathway analyses indicate altered cell adhesion, focal adhesion and cytoskeleton-related pathways. These changes may correlate with shifts in epithelial polarity and tissue architecture during OPA progression. Combined with the pathological observation results, this decline in molecular-level structural stability may be associated with the morphological changes in HE staining, such as the proliferation of AT2 cells and their protrusion into the alveolar cavity.

Endoplasmic reticulum stress is the core hub connecting viral infections and cell fate determination. GO enrichment analysis shows that the differentially expressed proteins are significantly enriched at the cellular component level in endoplasmic reticulum–Golgi complex-related membrane structures. “Protein processing in the endoplasmic reticulum” pathway is also significantly enriched in KEGG analysis (Figure 5B), overall suggesting that the endoplasmic reticulum homeostasis of the host cell undergoes systematic abnormalities after JSRV infection. The PPI network further confirmed that the core molecules of endoplasmic reticulum stress, such as HYOU1, SSR1, and SSR3, have strong interactive relationships and are core components of the regulatory network (Figure 5D). Viral infection is often accompanied by the synthesis and misfolding of a large number of viral proteins, which triggers the unfolded protein response (UPR) [28,29]. Moderate endoplasmic reticulum stress helps cells adapt to microenvironmental changes, whereas persistent or excessive stress triggers apoptosis [30]. The enrichment results suggest that endoplasmic reticulum stress-related processes may participate in OPA progression, and a continuous stress state may contribute to the metabolic reprogramming observed in lung tissues in this study, although the causal relationship remains to be further investigated [31].

Alterations in proteins associated with metabolic reprogramming and immune-inflammatory responses were observed concurrently and may represent an important molecular feature of JSRV infection. Among the top 50 differentially expressed proteins, proteins related to metabolism and oxidative stress homeostasis maintenance, such as FABP1, GGT1, and CAVIN3, were widely downregulated, suggesting that JSRV infection can trigger the remodelling of the lung tissue metabolic network at the protein level. These proteins maintain lipid metabolism homeostasis, antioxidant defence systems, and redox balance in alveolar type II epithelial cells (AT2 cells, the target cells of JSRV infection). Downregulation of FABP1 expression in hepatocellular carcinoma has been shown to upregulate the expression of proteins related to lipid metabolism synthesis, increase the level of cellular oxidative stress, and be associated with enhanced invasive behaviour and malignant phenotypes of tumour cells [32]; GGT1 is a reliable marker for assessing cellular dysfunction, and in breast cancer, GGT1 is significantly upregulated with a carcinogenic effect [33], contrary to the significant downregulation of GGT1 in the JSRV infection group in this study, suggesting that GGT1 may show functional heterogeneity in different tissue sources and different tumour-driving factors, and that JSRV infection may downregulate GGT1 to disrupt the antioxidant defence system of AT2 cells, exacerbate oxidative stress imbalance, which may contribute to epithelial cell transformation-associated molecular alterations. However, the underlying mechanisms require further verification. CAVIN3, a key tumour suppressor protein, is expressed at significantly low levels in lung cancer, breast cancer, and other malignant tumours and can exert tumour suppressor effects by inhibiting the PI3K/Akt signalling pathway and maintaining oxidative stress homeostasis [34,35], consistent with the study results. Thus, JSRV infection may downregulate CAVIN3, potentially relieving PI3K/Akt pathway inhibition and exacerbating oxidative stress imbalance, which may be associated with molecular changes linked to AT2 cell transformation.

In KEGG pathway analysis, significant enrichment of alanine/aspartate/glutamate metabolism and cytochrome P450-related pathways further supported the abnormality of amino acid and exogenous substance metabolism. The PPI network provides a more detailed regulatory picture of metabolic reprogramming. In addition to the endoplasmic reticulum stress module, the other core module of the network comprises metabolic and inflammatory-related proteins such as ASS1, GLS2, GFPT1, ARG1, and PTGS2, which mediate key pathways such as arginine metabolism, glutamine metabolism, and arachidonic acid metabolism, and are potential molecular nodes linking metabolic reprogramming with immune-inflammatory processes [36,37,38,39]. In breast cancer, high ARMC6 expression is associated with poor prognosis, and may contribute to tumour cell proliferation and immune regulatory alterations by regulating the CDC42 pathway and upregulating COX2 expression [40]. In ovarian cancer, AKAP2 anchors PKA to activate the β-catenin/TCF signalling pathway and upregulate target genes such as Snail and c-Myc, potentially contributing to cancer cell growth and migration [41]. This opposite expression pattern may reflect species-specific or virus-driven differences in tumorigenesis mechanisms.

This difference may stem from the heterogeneity of cross-species and tumour microenvironments: OPA tumours are mainly composed of AT2 cells, and their unique lipid metabolism microenvironment (downregulation of proteins such as FABP1 leading to oxidative stress) results in different dependencies on signalling pathways compared with human cancer cells—human breast cancer cells highly rely on the ARMC6-CDC42 axis to maintain cytoskeletal reorganisation and malignant proliferation [40], whereas in the specific stress microenvironment of OPA, loss of scaffold proteins such as AKAP2 may facilitate tumour cells to bypass the growth limitations of the classical PKA and Wnt pathways. Further, the core reason lies in the “pathway dependence” of the JSRV-specific oncogenic mechanism. Human spontaneous tumours need to accumulate various mutations to gradually activate the upstream proliferation pathways [40,41], whereas the envelope protein (Env) of JSRV itself has strong oncogenic activity and can independently activate the downstream proliferative network directly through host upstream signalling molecules [42,43]. Therefore, after JSRV induces tumour formation, the viral Env protein continuously activates downstream proliferative signals, and the tumour cells can selectively downregulate the original upstream or parallel oncogenic molecules (AKAP2 and ARMC6), which may simultaneously mediate negative regulatory functions such as cell differentiation, contact inhibition, and apoptosis; thus, their downregulation may provide a selective advantage under the JSRV-associated oncogenic environment.

Non-targeted metabolomics results confirmed the metabolic and inflammatory remodelling observed at the proteomic level. Chemical classification of the differential metabolites indicated that lipids and lipid-like molecules accounted for the highest proportion (33.91%), suggesting that lipid remodelling is the core metabolic feature of JSRV infection. KEGG enrichment analysis of the metabolome revealed four major abnormal pathways: (1) Widespread disturbances in amino acid metabolism represent the most prominent metabolic phenotype of JSRV infection. ABC transporters, protein digestion and absorption, and amino acid biosynthesis pathways are significantly enriched with an overall downward trend, providing a direct metabolic explanation for ARG1 and GFPT1 upregulation in the proteome: High ARG1 expression indicates a shift in arginine metabolism towards polyamine synthesis, potentially providing metabolic substrates that support increased cellular biosynthetic activity and inhibiting T-cell function by consuming arginine in the microenvironment, thus providing metabolic cues for forming the local immune-suppressive microenvironment of OPA [44,45]; GFPT1 upregulation can accelerate the amino transfer from glutamine to the amino acid hexose synthesis pathway [46,47,48,49]; this metabolic pattern is consistent with features reported in rapidly proliferating and transformed cells owing to their high dependence on glutamine metabolism to support rapid proliferation [49]. Thus, under the JSRV Env-driven activation of downstream proliferative signals (such as PI3K/Akt/mTOR), the demand for amino acids and other biological macromolecular precursors by tumour cells increases significantly, leading to the extensive consumption of free amino acids within the tissue. This confirms the downregulation of metabolic homeostasis maintenance proteins such as GGT1 in the proteome and reveals the metabolic pattern by which tumour cells consume host nutrient resources. (2) The arachidonic acid metabolism pathway was significantly enriched, providing key metabolic evidence for the local inflammatory immune state in OPA. Arachidonic acid is a direct substrate in the catalytic synthesis of prostaglandin E2 (PGE2) by PTGS2 (COX-2) [50]. The overall activation of this pathway was highly consistent with the significant upregulation of PTGS2 protein, reflecting the active pro-inflammatory lipid signalling cascade in OPA tumour tissues and its abnormal interweaving with the central carbon metabolism (TCA cycle) pathway, jointly representing a metabolic signalling pattern consistent with tumour-associated metabolic reprogramming. This discovery also provides a new perspective for the paradoxical phenomenon of “high expression of PGE2 synthase and low accumulation of intracellular products”. The activation of arachidonic acid metabolism may reflect altered prostaglandin and lipid mediator metabolism in OPA tissues. Although PTGS2 upregulation and metabolomic alterations are consistent with enhanced inflammatory lipid signalling, the precise role of PGE2 and related lipid mediators in shaping the OPA tumour microenvironment remains to be determined. These findings suggest a potential association between arachidonic acid-mediated lipid metabolism and carbon metabolism reprogramming. However, the specific contributions of these metabolic alterations to tumour microenvironmental regulation remain to be clarified. (3) Abnormal carbon metabolism pathways in the cancer centre indicate that the energy metabolism network of the lung tissue undergoes adaptive changes. The TCA cycle is a core hub of cellular energy metabolism and biomolecule synthesis [51,52], and abnormal key intermediate products suggest that under the continuous proliferative signal driven by JSRV Env protein activation, the observed alterations in TCA cycle intermediates may reflect adaptive changes in cellular energy metabolism associated with increased biosynthetic demands during OPA development. (4) Abnormal neural activity, ligand–receptor interactions, and serotonergic synapse pathways reveal a nonclassical regulatory dimension of metabolic reprogramming [53,54]. Metabolomics revealed significant disorders of neuroregulatory small molecules, such as tryptophan derivatives and lipid signalling molecules, and existing studies confirmed that neurotransmitters in the tumour microenvironment can directly regulate the chemotactic activity of tumour immune cells and the EMT process through paracrine pathways [55], suggesting the potential involvement of neuroimmune-associated metabolic processes in OPA. However, the functional significance of these alterations and their relationship to local immune regulation require further investigation.

The combined proteomic and metabolomic analyses further confirmed the synergistic regulatory relationship between the three core molecular programmes at a statistical level. The O2PLS model showed a highly coordinated molecular variation pattern between the two omics approaches (R2X = 0.968, R2Y = 0.878), confirming the intrinsic consistency of protein expression changes driven by JSRV infection and metabolite level changes. Common pathway analysis revealed pathways significantly enriched in both omics, including arachidonic acid metabolism, multiple amino acid metabolism, glutathione metabolism, and one-carbon unit metabolism, collectively indicating integrated changes in inflammatory lipid signalling, amino acid metabolism, redox regulation, and stress-related cellular processes. The co-pathway analysis further revealed molecular interaction relationships within the common pathways: with |r| > 0.8 and *p* < 0.05 as the threshold, significant correlations were identified between key proteins such as GFPT1, PTGS2, and RAC1 and metabolic nodes such as succinate, PGE2, and 1-hydroxyethylphosphatidylcholine, among which succinate, PGE2, and 1-hydroxyethylphosphatidylcholine are the core metabolic nodes connecting the proteomic remodelling and metabolomic changes. Notably, the co-network analysis identified a negative correlation between PTGS2 and PGE2. This association may reflect altered prostaglandin metabolism in OPA tissues; however, the underlying regulatory mechanisms and their potential implications for tumour microenvironmental changes remain unclear and require further investigation.

The expression levels of key node proteins in the multi-omics collaborative network were verified using Western blot analysis. Compared with the NC group, the expression levels of GFPT1, PTGS2, and ARG1 in the JSRV group were significantly increased, which is consistent with the quantitative proteomics results. These three proteins correspond to the aspartic acid sugar synthesis pathway, arachidonic acid—prostaglandin signalling axis, and arginine—polyamine metabolic branch, covering the three major core metabolic reprogramming directions identified in this study. This further verifies the authenticity and reliability of the cross-omics collaborative regulatory network at the experimental level.

Compared with traditional genetically engineered mouse models, OPA, a naturally occurring large animal tumour model, has irreplaceable advantages in terms of tumour heterogeneity, immune microenvironment complexity, and imaging assessment feasibility [16,17,18]. The protein–metabolism collaborative regulatory map drawn in this study provides a new perspective for understanding OPA pathogenesis and provides candidate molecules that may warrant future investigation in human lung adenocarcinoma studies (especially early lung adenocarcinoma with negative driver genes), such as GFPT1, PTGS2, and ARG1, which are key metabolic and immune regulatory molecules. However, the direct functional roles of GFPT1, PTGS2, and ARG1 in OPA pathogenesis remain to be elucidated and require further validation through gene manipulation and mechanistic studies.

This study has certain limitations. First, as an exploratory atlas study, the sample size was relatively small (*n* = 3). Although strict quality control procedures and non-parametric statistical analyses were applied to improve the robustness of the dataset, validation in larger independent cohorts will be necessary to further strengthen the generalizability of these findings. In addition, independent validation cohorts were not available in the present study. Second, this study adopted a strategy of comparing lesion areas with corresponding healthy tissues, which can capture the global characteristics of tumour and microenvironment cell communication; however, it is difficult to precisely attribute specific molecular changes to a single cell type. In the future, spatial multi-omics techniques need to be combined to complete the precise dissection of cell sources. Third, the protein–metabolite interactions revealed by the association network are mainly statistical correlations. Although GFPT1, PTGS2, and ARG1 were validated at the protein expression level by Western blotting, broader orthogonal validation approaches were not available in the present study, and their direct functional roles and causal regulatory relationships in OPA pathogenesis remain to be verified through gain- and loss-of-function studies, targeted proteomic and metabolomic analyses, and additional independent validation cohorts. Fourth, although previous studies have established alveolar type II pneumocytes and Clara/club cells as the principal target cells of JSRV infection and major components of OPA lesions, the present analyses were conducted using whole-lesion tissues. Consequently, some molecular signals may also reflect contributions from stromal and immune cell populations. Future spatial omics and single-cell studies will be valuable for resolving cell-type-specific molecular alterations.

## 5. Conclusions

In summary, through an integrated analysis of proteomics and non-targeted metabolomics, this study provides, for the first time, an integrated overview of proteomic and metabolomic alterations associated with JSRV-induced ovine pulmonary adenocarcinoma, with adhesion remodelling as the basis of cell morphology and endoplasmic reticulum stress as the intracellular signal-sensing hub, both associated with alterations in amino acid and lipid metabolic reprogramming as well as immune microenvironmental changes, potentially contributing to OPA pathogenesis. These findings provide a resource for future mechanistic studies of retrovirus-associated tumorigenesis and may facilitate the identification of candidate pathways for further investigation.

## Figures and Tables

**Figure 1:**
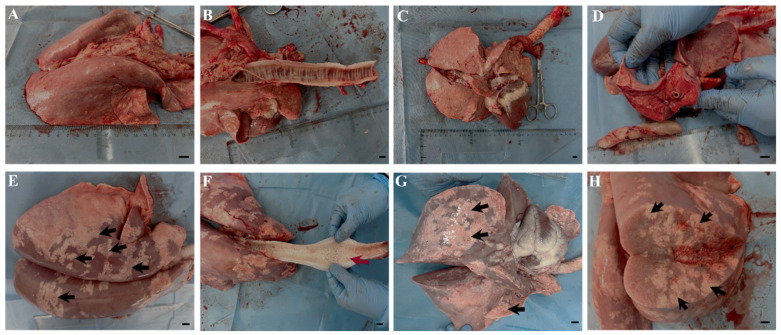
Pathological examination results. (**A**–**D**) Lung tissues from the negative control sheep (sheep No. 1), showing morphological characteristics consistent with healthy lung tissue. (**E**–**H**) Gross observation of lung tissues from JSRV-infected sheep (sheep No. 5, experimental group). Black arrows indicate diffuse grayish-white nodules on the surface of lung tissues, which are typical macroscopic lesions of OPA; red arrows indicate white mucus present in the trachea. Scale bar: 1 cm.

**Figure 2:**
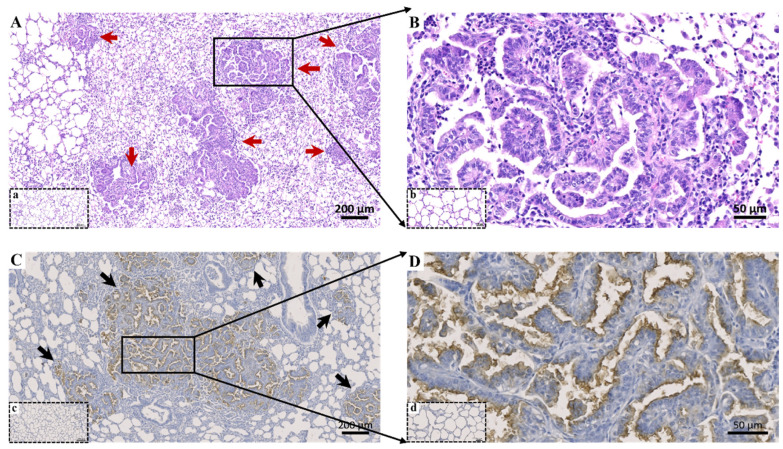
Histopathological and immunohistochemical verification of the JSRV-induced OPA animal model in sheep. (**A**,**B**) Haematoxylin and eosin (HE) staining images of the lung tissue from sheep in the JSRV infection group (sheep No. 5), with significant proliferation of type II alveolar epithelial cells (red arrows), scale: 200 μm and 50 μm. (**a**,**b**) HE staining images of the lung tissue from negative control group sheep (sheep No. 1), scale: 200 μm. (**C**,**D**) Immunohistochemical results of the lung tissue from sheep in the JSRV infection group (sheep No. 5), showing strong positive staining with JSRV Env polyclonal antibody in the adenocarcinoma lesion area (black arrows), scale: 200 μm and 50 μm. (**c**,**d**) Immunohistochemical results of the lung tissue from the negative control group sheep (sheep No. 1), scale: 200 μm.

**Figure 3:**
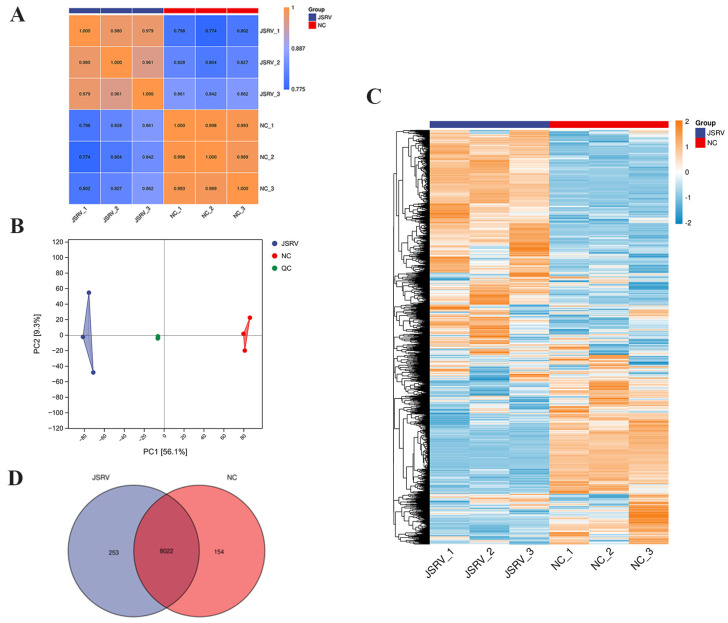
Quality control and overall expression profile analysis of proteomic data from lung tissues of sheep in the JSRV infection and negative control (NC) groups. (**A**) Pearson correlation heatmap showing pairwise correlation coefficients between all samples. The colour gradient (blue to orange) represents correlation strength (0.775−1.000). (**B**) Principal component analysis (PCA) score plot based on the total protein expression profile. PC1 accounts for 56.1% of the variance, and PC2 accounts for 9.3%. Blue dots = JSRV group, red dots = NC group, green dot = quality control (QC) sample. (**C**) Global hierarchical clustering heatmap of all quantified proteins. The colour bar (orange to blue) indicates normalised protein expression levels (2 to −2). Rows represent proteins, columns represent samples, with dendrograms showing clustering relationships. (**D**) Venn diagram illustrating the overlap of quantified proteins between the JSRV and NC groups. A total of 8022 proteins were shared between groups, while 253 and 154 proteins were uniquely detected in the JSRV and NC groups, respectively.

**Figure 4:**
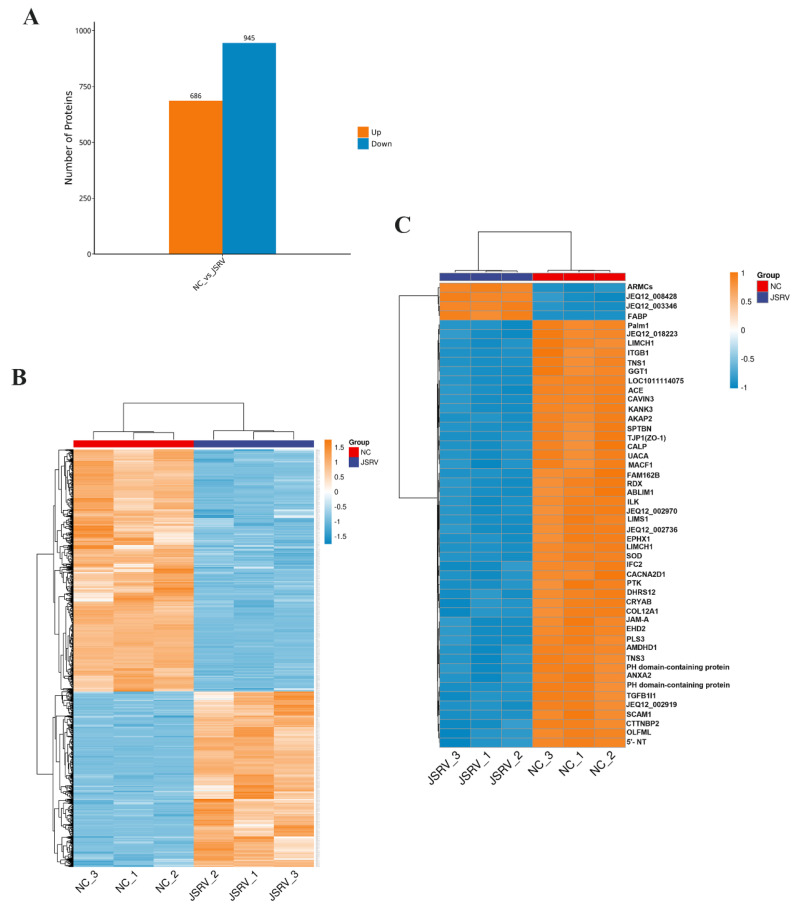
Screening and hierarchical clustering analysis of differentially expressed proteins (DEPs) induced by JSRV infection. (**A**) Statistical bar chart of DEPs between the negative control (NC) and JSRV groups. Orange bars represent upregulated proteins (*n* = 686) and blue bars represent downregulated proteins (*n* = 945). (**B**) Global hierarchical clustering heatmap of all identified DEPs. Rows represent proteins, columns represent samples from the NC and JSRV groups. The colour bar indicates normalised protein expression levels (1 to −1). (**C**) Hierarchical clustering heatmap of the top 50 most significantly DEPs. Rows represent proteins (labelled on the right), columns represent samples from the two groups. The colour bar indicates normalised protein expression levels (1.5 to −1.5).

**Figure 5:**
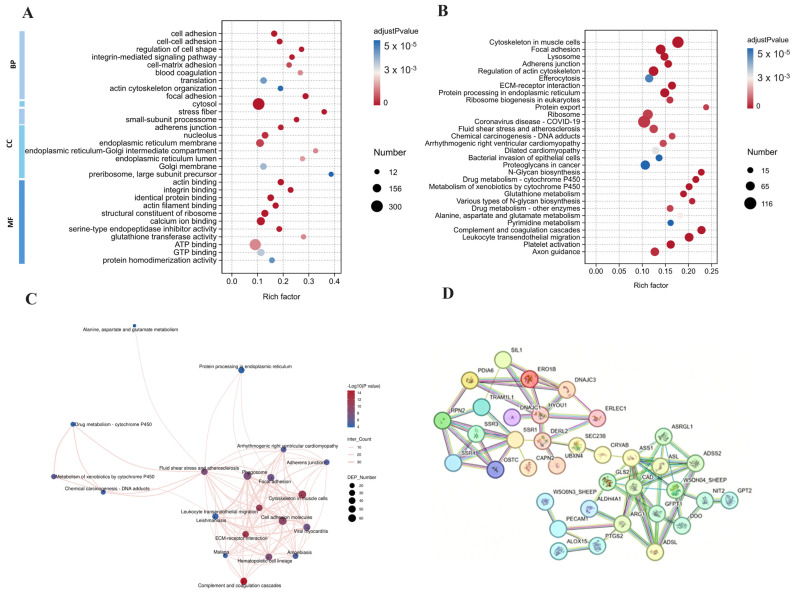
Functional enrichment analysis of differentially expressed proteins (DEPs) and construction of core regulatory protein interaction networks. (**A**) Bubble plot of the top 30 Gene Ontology (GO) functional enrichment terms for DEPs, categorised into biological process (BP), cellular component (CC), and molecular function (MF). The *x*-axis represents the rich factor, the *y*-axis represents GO terms, the colour gradient represents adjusted *p* values (blue to red, from small to large), and the bubble size represents the number of enriched proteins. (**B**) Bubble plot of Kyoto Encyclopedia of Genes and Genomes (KEGG) pathway enrichment for DEPs. The *x*-axis represents the rich factor, the *y*-axis represents pathway names, the colour gradient represents adjusted *p* values, and the bubble size represents the number of enriched proteins. (**C**) KEGG pathway enrichment network diagram. Nodes represent enriched pathways, with node colour indicating −log10 (*p* value) and node size indicating the number of enriched DEPs. Lines represent associations between pathways. (**D**) Protein–protein interaction (PPI) network of DEPs. Nodes represent proteins, with node size indicating protein connectivity, and line thickness indicating the strength of the interaction.

**Figure 6:**
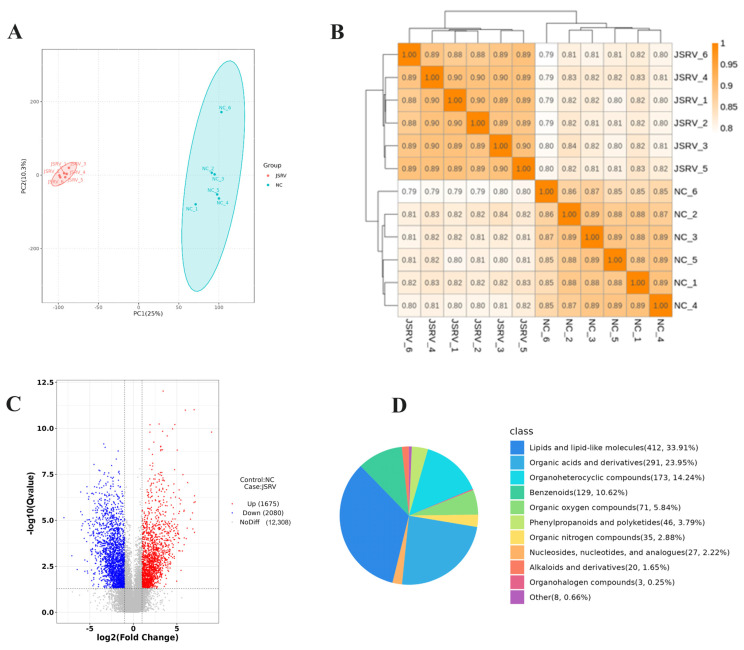
Quality control and overall characteristic analysis of metabolomics data from lung tissues of sheep in the JSRV infection group and negative control (NC) group. (**A**) Principal component analysis (PCA) score plot based on the expression profiles of all metabolites. Red dots represent the JSRV group, and cyan dots represent the NC group. PC1 accounts for 25% of the variance, and PC2 accounts for 10.3% of the variance, showing clear separation between the two groups. (**B**) Pearson correlation heatmap of all samples. The orange gradient represents correlation coefficients (ranging from 0.8 to 1.0). Intra-group correlation coefficients are all greater than 0.8, confirming high biological reproducibility within groups. (**C**) Volcano plot of differential metabolites between the JSRV and NC groups. The *x*-axis represents log_2_(Fold Change), and the *y*-axis represents − log_10_(Q value). Red dots indicate upregulated metabolites (*n* = 1675), blue dots indicate downregulated metabolites (*n* = 2080), and grey dots indicate non-differential metabolites (*n* = 12,308). (**D**) Pie chart showing the chemical classification of all identified metabolites. The main categories include lipids and lipid-like molecules (412, 33.91%), organic acids and derivatives (291, 23.95%), organoheterocyclic compounds (173, 14.24%), and other classes, with counts and percentages labelled for each category.

**Figure 7:**
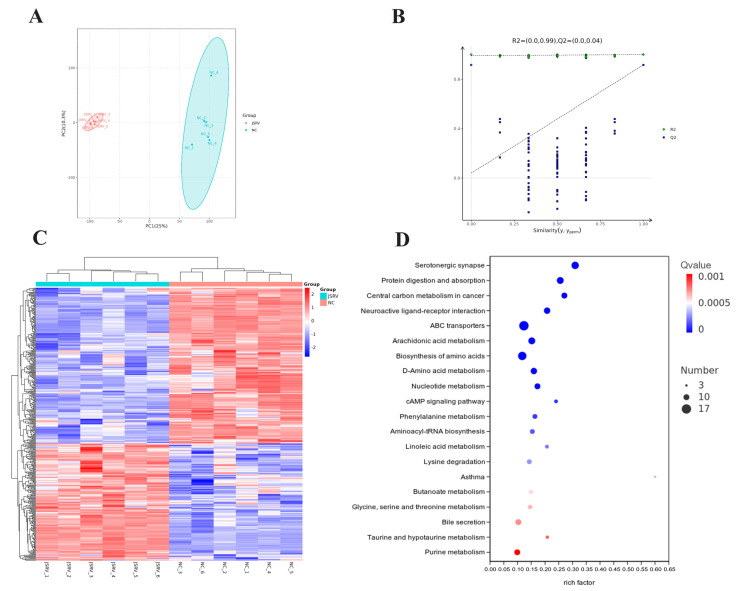
Screening and functional enrichment analysis of differential metabolites between the JSRV infection group and negative control (NC) group. (**A**) Principal component analysis (PCA) score plot based on the total metabolite expression profile. Red dots represent the JSRV group, and cyan dots represent the NC group. PC1 accounts for 25% of the variance, and PC2 accounts for 10.3% of the variance, showing clear separation between the two groups. (**B**) Permutation test result of the Orthogonal Partial Least Squares Discriminant Analysis (OPLS-DA) model. The green points represent R^2^ values, and the blue points represent Q^2^ values (R^2^ = (0.0, 0.99), Q^2^ = (0.0, 0.04)), indicating no overfitting risk of the model. (**C**) Hierarchical clustering heatmap of differential metabolites. Rows represent metabolites, columns represent samples from the JSRV and NC groups. The colour gradient (red to blue) indicates normalised metabolite expression levels (high to low). (**D**) Bubble plot of KEGG pathway enrichment for differential metabolites. The *x*-axis represents the rich factor, the *y*−axis represents pathway names, the colour gradient represents Q values (from 0 to 0.001), and the bubble size represents the number of enriched metabolites.

**Figure 8:**
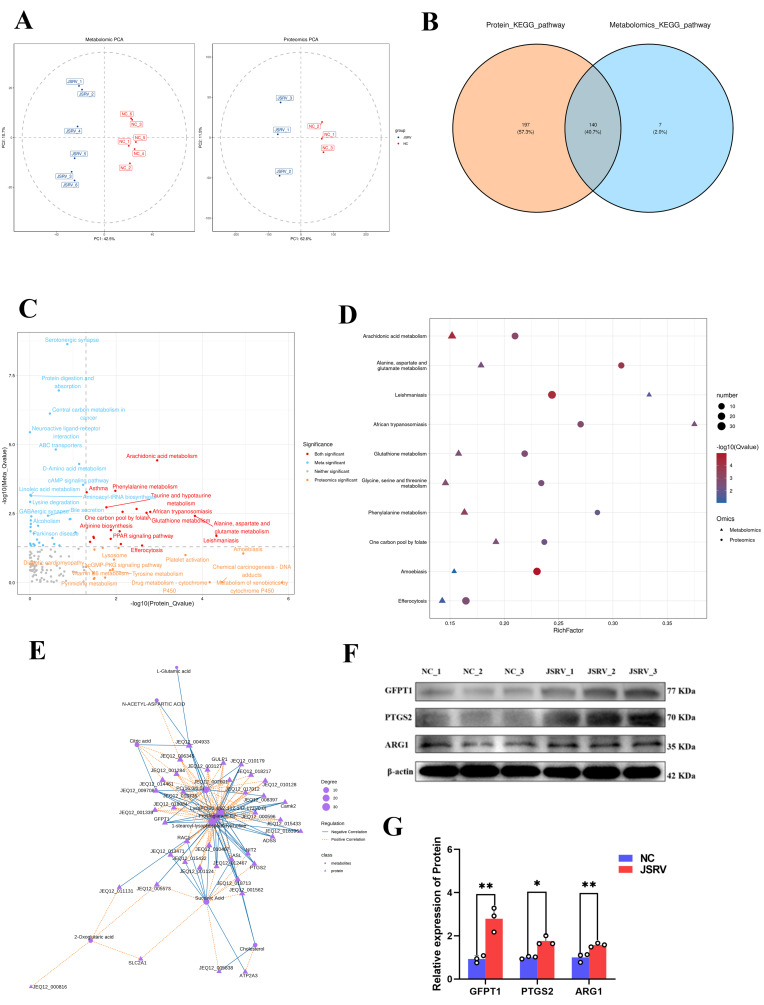
Multi-omics integration analysis and validation of key proteins in JSRV-induced OPA. (**A**) PCA score plots for metabolome (left) and proteome (right) expression profiles. (**B**) Venn diagram of shared and unique KEGG pathways enriched by the proteome and metabolome. (**C**) Scatter plot comparing KEGG pathway enrichment results between the proteome and metabolome. (**D**) Bubble plot of co-enriched KEGG pathways across the two omics datasets. (**E**) Correlation network diagram of key proteins and metabolites (|r| > 0.8, *p* < 0.05). (**F**) Western blot bands of key proteins (GFPT1, PTGS2, ARG1) in the JSRV and NC groups, with β−actin as the internal reference. (**G**) Relative expression levels of key proteins, with statistical significance marked (* *p* < 0.05, ** *p* < 0.01).

## Data Availability

The datasets presented in this study can be found in online repositories. The names of the repository/repositories and accession number(s) can be found below: Metabolome Database (OMIX) of the National Genomics Data Center (NGDC). The accession numbers are OMIX017069 (metabolomics dataset) and OMIX017070 (proteomics dataset).

## Supplementary Materials

The following supporting information can be downloaded at: https://www.mdpi.com/article/10.3390/biology15130982/s1, Figure S1: Quality control analysis results; Figure S2: TIC Overlay Plot of QC Samples.

## References

[1] Griffiths D.J., Martineau H.M., Cousens C. (2010). Pathology and Pathogenesis of Ovine Pulmonary Adenocarcinoma. J. Comp. Pathol..

[2] Cousens C., Thonur L., Imlach S., Crawford J., Sales J., Griffiths D.J. (2009). Jaagsiekte Sheep Retrovirus Is Present at High Concentration in Lung Fluid Produced by Ovine Pulmonary Adenocarcinoma-Affected Sheep and Can Survive for Several Weeks at Ambient Temperatures. Res. Vet. Sci..

[3] Caporale M., Cousens C., Centorame P., Pinoni C., Heras M.D.L., Palmarini M. (2006). Expression of the Jaagsiekte Sheep Retrovirus Envelope Glycoprotein Is Sufficient to Induce Lung Tumors in Sheep. J. Virol..

[4] Cousens C., Maeda N., Murgia C., Dagleish M.P., Palmarini M., Fan H. (2007). In Vivo Tumorigenesis by Jaagsiekte Sheep Retrovirus (JSRV) Requires Y590 in Env TM, but Not Full-Length orfX Open Reading Frame. Virology.

[5] Palmarini M., Maeda N., Murgia C., De-Fraja C., Hofacre A., Fan H. (2001). A Phosphatidylinositol 3-Kinase Docking Site in the Cytoplasmic Tail of the Jaagsiekte Sheep Retrovirus Transmembrane Protein Is Essential for Envelope-Induced Transformation of NIH 3T3 Cells. J. Virol..

[6] Martineau H.M., Cousens C., Imlach S., Dagleish M.P., Griffiths D.J. (2011). Jaagsiekte Sheep Retrovirus Infects Multiple Cell Types in the Ovine Lung. J. Virol..

[7] Liu S.L., Lerman M.I., Miller A.D. (2003). Putative Phosphatidylinositol 3-Kinase (PI3K) Binding Motifs in Ovine Betaretrovirus Env Proteins Are Not Essential for Rodent Fibroblast Transformation and PI3K/Akt Activation. J. Virol..

[8] de las Heras M., Ortín A., Benito A., Summers C., Ferrer L., Sharp J. (2006). In Situ Demonstration of Mitogen-Activated Protein Kinase Erk 1/2 Signalling Pathway in Contagious Respiratory Tumours of Sheep and Goats. J. Comp. Pathol..

[9] Summers C., Dewar P., van der Molen R., Cousens C., Salvatori D., Sharp J.M., Griffiths D.J., Norval M. (2006). Jaagsiekte Sheep Retrovirus-Specific Immune Responses Induced by Vaccination: A Comparison of Immunisation Strategies. Vaccine.

[10] Summers C., Neill W., Dewar P., Gonzalez L., van der Molen R., Norval M., Sharp J.M. (2002). Systemic immune responses following infection with Jaagsiekte sheep retrovirus and in the terminal stages of ovine pulmonary adenocarcinoma. J. Gen. Virol..

[11] Caporale M., Martineau H., de las Heras M., Murgia C., Huang R., Centorame P., Di Francesco G., Di Gialleonardo L., Spencer T.E., Griffiths D.J. (2013). Host Species Barriers to Jaagsiekte Sheep Retrovirus Replication and Carcinogenesis. J. Virol..

[12] Palmarini M., Mura M., Spencer T.E. (2004). Endogenous Betaretroviruses of Sheep: Teaching New Lessons in Retroviral Interference and Adaptation. J. Gen. Virol..

[13] Karagianni A.E., Vasoya D., Finlayson J., Martineau H.M., Wood A.R., Cousens C., Dagleish M.P., Watson M., Griffiths D.J. (2019). Transcriptional Response of Ovine Lung to Infection with Jaagsiekte Sheep Retrovirus. J. Virol..

[14] Youssef G., Wallace W.A.H., Dagleish M.P., Cousens C., Griffiths D.J. (2015). Ovine Pulmonary Adenocarcinoma: A Large Animal Model for Human Lung Cancer. ILAR J..

[15] Gray M.E., Meehan J., Sullivan P., Marland J.R.K., Greenhalgh S.N., Gregson R., Clutton R.E., Ward C., Cousens C., Griffiths D.J. (2019). Ovine Pulmonary Adenocarcinoma: A Unique Model to Improve Lung Cancer Research. Front. Oncol..

[16] Palmarini M., Fan H. (2001). Retrovirus-Induced Ovine Pulmonary Adenocarcinoma, an Animal Model for Lung Cancer. J. Natl. Cancer Inst..

[17] Gray M.E., Sullivan P., Marland J.R.K., Greenhalgh S.N., Meehan J., Gregson R., Clutton R.E., Cousens C., Griffiths D.J., Murray A. (2019). A Novel Translational Ovine Pulmonary Adenocarcinoma Model for Human Lung Cancer. Front. Oncol..

[18] Scott P.R., Dagleish M.P., Cousens C. (2018). Development of Superficial Lung Lesions Monitored on Farm by Serial Ultrasonographic Examination in Sheep with Lesions Confirmed as Ovine Pulmonary Adenocarcinoma at Necropsy. Ir. Vet. J..

[19] Palmarini M., Sharp J.M., de las Heras M., Fan H. (1999). Jaagsiekte Sheep Retrovirus Is Necessary and Sufficient to Induce a Contagious Lung Cancer in Sheep. J. Virol..

[20] Cousens C., Meehan J., Collie D., Wright S., Chang Z., Todd H., Moore J., Grant L., Daniel C.R., Tennant P. (2024). Tracking Ovine Pulmonary Adenocarcinoma Development Using an Experimental Jaagsiekte Sheep Retrovirus Infection Model. Genes.

[21] Zhang P., Wang Y., Duan X., Chen S., Du X., Bao A., Ma X., Zhang Y., Liu S. (2025). Exogenous Jaagsiekte Sheep Retrovirus (JSRV) Inner Mongolia Strain: Whole-Genome Characterization and Viral Particle Packaging. Front. Vet. Sci..

[22] Kim D.H., Lee C.W., Choi Y.J., Kim D.-S., Ban K., Hong J., Moon G.J., Kim S.-Y., Pack C.-G., Baek I.-J. (2026). Tumor Exosomal L1 Cell Adhesion Molecule Promotes Brain Metastasis of Lung Cancer. Research.

[23] Karlsson T., Kvarnbrink S., Holmlund C., Botling J., Micke P., Henriksson R., Johansson M., Hedman H. (2018). LMO7 and LIMCH1 Interact with LRIG Proteins in Lung Cancer, with Prognostic Implications for Early-Stage Disease. Lung Cancer.

[24] Zhu S., Cheng Z., Wu Z., Wang J. (2022). High Impact of ITGB1 on PI3K/AKT Expression in Medulloblastoma. Neuro Oncol..

[25] Wang Z., Ye J., Dong F., Cao L., Wang M., Sun G. (2022). TNS1: Emerging Insights into Its Domain Function, Biological Roles, and Tumors. Biology.

[26] Chen L., Ren Z., Cai Y. (2021). Construction and Analysis of Survival-Associated Competing Endogenous RNA Network in Lung Adenocarcinoma. Biomed. Res. Int..

[27] Martuszewska D., Ljungberg B., Johansson M., Landberg G., Oslakovic C., Dahlbäck B., Hafizi S. (2009). Tensin3 Is a Negative Regulator of Cell Migration and All Four Tensin Family Members Are Downregulated in Human Kidney Cancer. PLoS ONE.

[28] Wei S., Zhang N., Zhang H., Chen Z., Li S., Wu W., Liu Z., Xia Z., Luo P., Cheng Q. (2026). Endoplasmic Reticulum Stress in Disease Pathogenesis: Its Implications for Therapy. Signal Transduct. Target. Ther..

[29] Prasad V., Greber U.F. (2021). The Endoplasmic Reticulum Unfolded Protein Response–Homeostasis, Cell Death and Evolution in Virus Infections. FEMS Microbiol. Rev..

[30] Cirone M. (2021). ER Stress, UPR Activation and the Inflammatory Response to Viral Infection. Viruses.

[31] Wu Y., Luo H., Pan Z., Chen W., Bi L. (2025). Bidirectional Crosstalk Between ER Stress and Lipid Metabolism: From Proteostasis to Tumor Adaptation. Cell Death Discov..

[32] Lin Y.-X., Wu X.-B., Zheng C.-W., Zhang Q.-L., Zhang G.-Q., Chen K., Zhan Q., An F.-M. (2021). Mechanistic Investigation on the Regulation of FABP1 by the IL-6/miR-603 Signaling in the Pathogenesis of Hepatocellular Carcinoma. Biomed. Res. Int..

[33] Coradini D., Gambazza S., Oriana S., Ambrogi F. (2020). Body Mass Index and γ-Glutamyl Transferase Expression in Normal and Cancerous Breast Tissue. Breast Cancer.

[34] An X., Lin X., Yang A., Jiang Q., Geng B., Huang M., Lu J., Xiang Z., Yuan Z., Wang S. (2020). Cavin3 Suppresses Breast Cancer Metastasis via Inhibiting AKT Pathway. Front. Pharmacol..

[35] Qian L., Zhang Z.Q., Cai H.N. (2025). The Role of Cavin3 in the Development of Malignant Tumors. Cancer Manag. Res..

[36] Dias M.M., Adamoski D., Dos Reis L.M., Ascenção C.F.R., de Oliveira K.R.S., Mafra A.C.P., da Silva Bastos A.C., Quintero M., Cassago C.d.G., Ferreira I.M. (2020). GLS2 is protumorigenic in breast cancers. Oncogene.

[37] Tang W., Gao Y., Hong S., Wang S. (2024). GFPT1 accelerates immune escape in breast cancer by modifying PD-L1 via O-glycosylation. BMC Cancer.

[38] Ma X., Huo Z., Yang T., Xiao J., Zhao Y., Gong N., Liu G., Wang H. (2023). Elucidating the molecular mechanisms underlying anti-inflammatory effects of Morchella esculenta in the arachidonic acid metabolic pathway by network pharmacology and molecular docking. Sci. Rep..

[39] Liu X., Ren B., Ren J., Gu M., You L., Zhao Y. (2024). The significant role of amino acid metabolic reprogramming in cancer. Cell Commun. Signal..

[40] Zhang R.X. (2024). On the Oncogenic Role and Molecular Mechanism of ARMC6 in Breast Cancer. Doctoral Dissertation.

[41] Li X., Wang C., Zhang G., Liang M., Zhang B. (2017). AKAP2 Is Upregulated in Ovarian Cancer, and Promotes Growth and Migration of Cancer Cells. Mol. Med. Rep..

[42] Liu S.L., Miller A.D. (2007). Oncogenic Transformation by the Jaagsiekte Sheep Retrovirus Envelope Protein. Oncogene.

[43] Maeda N., Fu W., Ortin A., de las Heras M., Fan H. (2005). Roles of the Ras-MEK-Mitogen-Activated Protein Kinase and Phosphatidylinositol 3-Kinase-Akt-mTOR Pathways in Jaagsiekte Sheep Retrovirus-Induced Transformation of Rodent Fibroblast and Epithelial Cell Lines. J. Virol..

[44] Sosnowska A., Chlebowska-Tuz J., Matryba P., Pilch Z., Greig A., Wolny A., Grzywa T.M., Rydzynska Z., Sokolowska O., Rygiel T.P. (2021). Inhibition of Arginase Modulates T-Cell Response in the Tumor Microenvironment of Lung Carcinoma. Oncoimmunology.

[45] Munder M. (2009). Arginase: An Emerging Key Player in the Mammalian Immune System. Br. J. Pharmacol..

[46] Wellen K.E., Lu C., Mancuso A., Lemons J.M., Ryczko M., Dennis J.W., Rabinowitz J.D., Coller H.A., Thompson C.B. (2010). The Hexosamine Biosynthetic Pathway Couples Growth Factor-Induced Glutamine Uptake to Glucose Metabolism. Genes Dev..

[47] Drury J., Geisen M.E., Tessmann J.W., Rychahou P.G., Kelson C.O., He D., Wang C., Evers B.M., Zaytseva Y.Y. (2024). Overexpression of Fatty Acid Synthase Upregulates Glutamine-Fructose-6-Phosphate Transaminase 1 and O-Linked N-Acetylglucosamine Transferase to Increase O-GlcNAc Protein Glycosylation and Promote Colorectal Cancer Growth. Int. J. Mol. Sci..

[48] Yang J.-Y., Zhang R., Zhang Z.-R., Li S., Gong D.-A., Li C.-H., Chen C., Huang L.-Y., Huang A.-L., Tang N. (2025). GFAT1 Promotes the Progression of Hepatocellular Carcinoma via Enhancing the O-GlcNAcylation of VEZF1. Cell Death Dis..

[49] Ward P.S., Thompson C.B. (2012). Metabolic Reprogramming: A Cancer Hallmark Even Warburg Did Not Anticipate. Cancer Cell.

[50] Kaur A., Mishra V.K., Kaur G., Kaur M., Bansal M. (2026). Structure-Based Dual Interactions of New Molecular Hybrids of NSAIDs with Arachidonic Acid Binding Pockets of COX-2 and 5-LOX. J. Mol. Struct..

[51] Tufail M., Jiang C.H., Li N. (2024). Altered Metabolism in Cancer: Insights into Energy Pathways and Therapeutic Targets. Mol. Cancer.

[52] Ciccarone F., Vegliante R., Di Leo L., Ciriolo M.R. (2017). The TCA Cycle as a Bridge Between Oncometabolism and DNA Transactions in Cancer. Semin. Cancer Biol..

[53] Liu Q.Q., Dong Z.K., Wang Y.F., Jin W.L. (2025). Reprogramming Neural-Tumor Crosstalk: Emerging Therapeutic Dimensions and Targeting Strategies. Mil. Med. Res..

[54] Chen L., Huang S., Wu X., He W., Song M. (2024). Serotonin Signalling in Cancer: Emerging Mechanisms and Therapeutic Opportunities. Clin. Transl. Med..

[55] Aysit N., Altintas E., Koksalar Alkan F., Ozturk G., Korkaya H. (2026). The Neural Niche in Cancer: Mechanistic Insights into Tumor-Neuron-Immune Crosstalk and Therapeutic Opportunities. Front. Cell Dev. Biol..

