# Supplementary figures and images for: Integrated Proteomic and Metabolomic Analyses Characterise Molecular Alterations Associated with JSRV-Induced OPA
Source: Biology (Basel). 2026 Jun 23;15(13):982. doi: 10.3390/biology15130982 (PMC13359867; doi:10.3390/biology15130982)

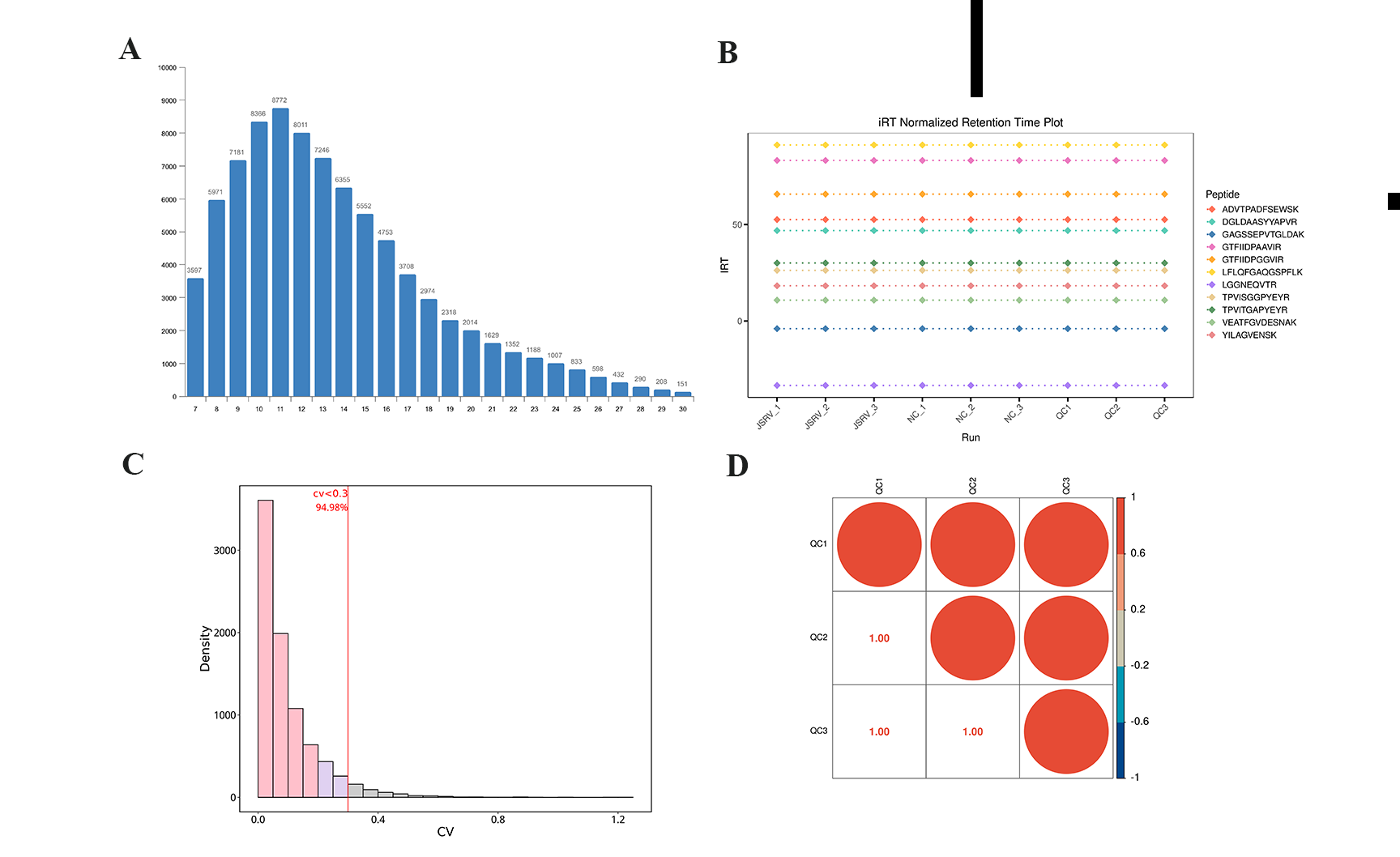

Supplement: Supplementary file 1 [file biology-15-00982-s001.zip › Supplementary Fig.S1.tif]

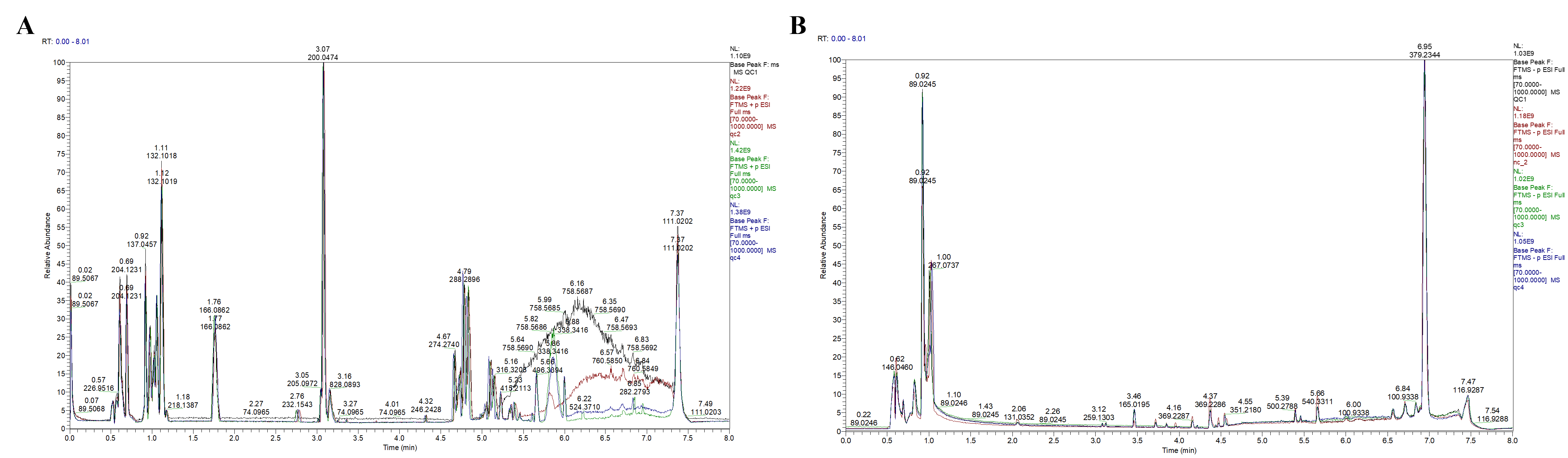

Supplement: Supplementary file 1 [file biology-15-00982-s001.zip › Supplementary Fig.S2.tif]
